# Harsh discipline mediates the association between parenting stress and internalizing problems in children and adolescents: survey-based and online intervention evidence

**DOI:** 10.3389/fpsyt.2026.1756447

**Published:** 2026-04-30

**Authors:** Xiaoxiao Wan, Ziyan Zhu, Xi Xuan, Yu Peng, Jiemin Yang

**Affiliations:** 1 Institute of Brain and Psychological Sciences, Sichuan Normal University, Chengdu, China; 2 Anhui Engineering and Technology Vocational School, Suzhou, China; 3 Mental Health Direction Center, Tianjin Railway Technical and Vocational College, Tianjin, China; 4 Zongbei West Middle School, Chengdu, China; 5 Sichuan Key Laboratory of Psychology and Behavior of Discipline Inspection and Supervision, Sichuan Normal University, Chengdu, China

**Keywords:** emotion regulation, harsh discipline, internalizing problems, online intervention, parenting stress

## Abstract

**Background:**

Parenting stress evokes harsh discipline and induces internalizing problems in children and adolescents. To test this hypothesis, this study examined the potential mediating role of harsh discipline in the association between parenting stress and internalizing problems in children and adolescents while considering the moderating effect of emotion regulation.

**Methods:**

Two studies were conducted: Study 1 was a cross-sectional survey using questionnaires (*N* = 971), and Study 2 implemented a three-week online parental intervention training program combining courses and psychological diary recording (*N* = 123).

**Results:**

Both studies consistently demonstrated that harsh discipline mediated the link between parenting stress and internalizing problems in children and adolescents. Furthermore, acceptance and cognitive reappraisal reduced the effect of parenting stress on harsh discipline, whereas distraction and rumination enhanced it. Expressive suppression had no significant moderating effect. The intervention enhanced parents’ emotion regulation (increased acceptance), reduced parenting stress and alleviated internalizing problems in children and adolescents, with preliminary evidence of reduced harsh discipline.

**Conclusion:**

These findings clarify the psychological mechanisms through which parenting stress influences child adaptiveness and underscore the value of interventions focused on emotion regulation in mitigating parenting stress, harsh discipline and enhancing child mental health.

## 1 Introduction

Epidemiological studies estimate that approximately 13.4% of children and adolescents worldwide experience clinically significant internalizing problems, with specific prevalence rates of 2.6% and 6.5% for depressive and anxiety disorders, respectively (1). Childhood and adolescence are critical developmental periods characterized by a high occurrence of internalizing and externalizing problem behaviors. Internalizing problems primarily involve disturbances in emotion regulation and psychological functioning, including symptoms of anxiety, withdrawal, and depression (2). Unlike their externalizing counterparts, internalizing problems are particularly insidious due to their covert nature and the tendency toward self-guilt and self-punishment (3). Research indicates that internalizing problems in children and adolescents can have a lasting influence on the individual, persisting throughout adolescence and even adulthood, leading to academic failure, suicide, and other adverse outcomes (4). Given these substantial negative outcomes, it is critical to identify risk factors and explore effective targeted interventions.

### 1.1 Conceptualizing internalizing problems: a developmental perspective

In this study, internalizing problems are operationalized as symptoms encompassing anxiety, depression, social withdrawal, and somatic complaints. Their manifestation evolves across development. In childhood, distress is often expressed through behavioral and somatic complaints, such as clinginess, avoidance, headaches, or stomachaches (5). Younger children have limited ability to report on mental health concerns (6), making parent reports more reliable at this age. In adolescence, with advances in cognitive and emotional maturity, problems manifest as pervasive negative mood, worthlessness, and maladaptive emotion regulation (7). Adolescents can accurately report their own emotional experiences, rendering self-report more informative during this period (8). Accordingly, the present research employed an age-appropriate, multi-informant assessment strategy: parent-report for children under 10, and self-report for adolescents aged 10 and above. This approach aligns with research indicating that pre-adolescence (around age 10) marks a critical transition in symptom manifestation and self-report capacity (9). Multiple factors influence internalizing problems in children and adolescents. Family systems theory emphasizes that family functioning is a major influence on child development (10). As two important aspects of family functioning, parenting stress and discipline behaviors are closely related to children’s problem behaviors.

### 1.2 Parenting stress as a key risk factor

Parenting stress refers to the stress that parents experience in the parenting role due to perceptions of dysfunctional parent-child interactions, challenging child behaviors, and associated role demands (11). It is usually independent of social and economic status and is related to parental role constraints, parent-child relationships, and child traits. According to the Family Stress Model (FSM), when individuals lack sufficient internal coping skills or external resources to manage stressors, the adverse effects of stress permeate the entire family system (12). Parents experiencing high levels of parenting stress are more likely to exhibit negative emotional expressions and maladaptive parenting behaviors, which may undermine children’s sense of security and increase the risk of internalizing problems such as depression and anxiety (13). Therefore, this study positions parenting stress as a key antecedent variable in the proposed model.

### 1.3 Harsh discipline as a mediator

Harsh parental discipline refers to coercive behaviors and negative emotional expressions that parents impose on their children (14). It encompasses a continuum ranging from minor psychological harm to serious injury and can be categorized into psychological aggression, corporal punishment, and physical abuse (15). In a survey of Chinese parents, over 70% reported using harsh discipline (16). However, research indicates that harsh discipline is associated with adverse child outcomes. Both psychological aggression and corporal punishment predict child depression (17, 18), and children experiencing harsher discipline show elevated anxiety and depression symptoms (19). These findings indicate that harsh discipline is a significant risk factor for internalizing problems. Furthermore, parenting stress is a robust predictor of harsh discipline. Parenting stress model posits that stress impairs parenting behaviors, leading to more negative and reactive styles (20). Empirical studies consistently show that under high stress, parents exhibit increased irritability and punitive responses, increasing their use of harsh discipline (21). In summary, these studies suggest that harsh discipline may mediate the effect of parenting stress on child internalizing problems, the core hypothesis of this study. However, the moderating mechanisms of this pathway remain unclear, particularly the conditions under which parenting stress is more likely to translate into harsh discipline and trigger child problems.

### 1.4 Emotion regulation as a moderator

Emotion regulation in parenting is recognized as an important protective factor (22). It refers to the process by which individuals influence the occurrence, experience, and expression of emotions. Its process model identifies five phases: situation selection, modification, attentional deployment, cognitive change, and response modulation (23). These stages involve distinct strategies: cognitive reappraisal and acceptance are considered adaptive, whereas distraction, expressive suppression, and rumination are considered maladaptive. These strategies may play different roles in parenting and warrant further investigation. The Family Stress Model (12) posits that the impact of stressors on parenting depends on parents’ internal coping resources. Emotion regulation constitutes a key coping resource that may influence whether stress-induced negative emotions escalate into punitive responses (22). Empirical evidence supports this perspective: whereas some parents under high stress resort to harsh discipline, others maintain supportive practices (24), indicating that parenting stress does not uniformly translate into harsh discipline but rather may depend on how parents regulate their emotions (25). Building on this theoretical and empirical foundation, the present study proposes that parental emotion regulation strategies moderate the association between parenting stress and harsh discipline, addressing the question of under what conditions the stress–discipline link is stronger or weaker.

Different emotion regulation strategies are theorized to have distinct moderating effects. Cognitive reappraisal involves reframing stressful parenting situations in non-emotional terms, reducing the emotional impact of parenting stress (26). Parents who habitually use this strategy may be better equipped to manage stress-induced negative emotions, potentially buffering the translation of stress into harsh discipline. Acceptance involves nonjudgmentally acknowledging stressful emotions rather than avoiding or suppressing them (27). By allowing emotions to exist naturally, acceptance may help prevent stress from escalating into explosive reactions, serving as a buffer. Conversely, maladaptive strategies are theorized to exacerbate the stress–discipline link. Rumination involves repetitively focusing on negative emotions and their causes (28). By amplifying negative emotions under stress, rumination may exacerbate the effect of parenting stress on harsh discipline. Distraction involves consciously removing attention from emotional situations (29). However, overreliance on distraction may mask root causes of emotional problems; under high parenting stress, distraction may fail to address underlying stressors, potentially enhancing the stress–discipline link. Expressive suppression involves inhibiting the outward manifestation of internal emotions (30). Under chronic parenting stress, prolonged suppression may deplete cognitive resources, potentially exacerbating the stress–discipline link (31). Identifying which emotion regulation strategies buffer or exacerbate this association is, therefore, critical for developing targeted interventions that can disrupt the pathway from parenting stress to harsh discipline.

### 1.5 Online parenting intervention training

Given that specific emotion regulation strategies may differentially moderate the association between parenting stress and harsh discipline, these strategies may represent important targets for intervention. Enhancing adaptive strategies may help parents respond more constructively under stress, thereby potentially disrupting the pathway from parenting stress to harsh discipline and subsequent internalizing problems in children and adolescents. Parenting programs are structured interventions that aim to improve parenting practices and reduce children’s behavioral and emotional difficulties (32). In recent years, numerous programs originally designed for in-person delivery have been adapted for online delivery, driven by technological advancements and accelerated by the COVID-19 pandemic (33). Compared to in-person delivery, online parenting programs offer advantages such as more flexible scheduling and improved accessibility (34). Meta-analytic evidence supports their efficacy in reducing child behavioral problems, enhancing parenting behavior and self-efficacy, alleviating parents’ mental health issues, and improving parent-child relationships (35).

### 1.6 The present study: an integrated moderated mediation model

Taken together, this research examines a moderated mediation model in which harsh discipline mediates the relationship between parenting stress and internalizing problems in children and adolescents, and specific parental emotion regulation strategies (cognitive reappraisal, acceptance, rumination, distraction, and expressive suppression) moderate the association between parenting stress and harsh discipline. To test this model, Study 1 employed a cross-sectional survey design to examine these moderating and mediating mechanisms. Building on these findings, Study 2 employed an online intervention design to evaluate whether targeting the adaptive strategies identified as protective moderators (cognitive reappraisal and acceptance) can attenuate parenting stress, harsh discipline, and internalizing problems in children and adolescents. Specifically, the intervention was designed to enhance parents’ use of these adaptive strategies, foster positive parenting skills, and thereby improve parent-child relationships. Building on Study 1, Study 2 targets the identified protective moderators, shifting from mechanism identification to experimental testing of their modifiability.

Based on the theoretical framework outlined above, the following hypotheses were tested. In Study 1: H1: Parenting stress will be positively associated with internalizing problems in children and adolescents, and harsh discipline will mediate this relationship. H2: Cognitive reappraisal and acceptance will buffer the association between parenting stress and harsh discipline. H3: Rumination, distraction, and expressive suppression will exacerbate the association between parenting stress and harsh discipline. In Study 2: H4: Compared to a control group, parents in the online intervention will show greater increases in adaptive emotion regulation strategies (cognitive reappraisal and acceptance) at post-intervention. H5: Compared to a control group, parents in the online intervention will show greater reductions in parenting stress, harsh discipline, and internalizing problems in children and adolescents at post-intervention.

## 2 Study 1

### 2.1 Participants and procedure

Participants were recruited through the Wenjuanxing proprietary panel database and targeted social media advertisements. The Wenjuanxing panel service distributed the questionnaire to a stratified sample of parents, ensuring representation across major geographic regions. To enhance demographic diversity, the survey link was also promoted on widely used social networking platforms in China, including WeChat Moments, Weibo, and Xiaohongshu, targeting users interested in parenting topics. All recruitment materials explicitly stated the eligibility criteria. Inclusion criteria were: (a) being a parent of a child currently enrolled in elementary through high school (approximately 6–18 years of age); (b) having regular internet access to complete online questionnaires; (c) ability to read and understand Chinese. Exclusion criteria were: (a) diagnosed severe mental illness in parents; (b) diagnosed developmental disorder or intellectual disability in the target child.

Study 1 distributed 1,200 questionnaires. After excluding invalid responses (e.g., those with brief completion times or incorrect answers to lie-detection items), 971 valid questionnaires were retained (51.0% female). Among these, 851 participants provided age information (Mage = 34.08, SD = 5.66). The sample consisted of 68.8% urban and 31.2% rural residents. Most participants (65.0%) had one child, while 35.0% had two or more. In terms of educational background, 35.4% held qualifications below a bachelor’s degree, and 64.6% possessed a bachelor’s degree or higher. Family structures included two-parent households (62.9%), single-parent households (0.4%), and other arrangements (36.7%, such as living with grandparents or relatives). Additionally, 62.1% reported grandparental involvement in child-rearing.

This study employed an online questionnaire to collect data. Participants provided demographic information and completed child-related assessments, with the procedure lasting 10–25 minutes. Before participation, researchers informed participants of the study’s minimal-risk nature, guaranteed data confidentiality for strictly academic use, and emphasized the voluntary nature of their participation. All participants provided informed consent, retaining the unconditional right to withdraw at any time. The data collection protocol received ethical approval from the psychological research ethics committee at the corresponding author’s institution.

### 2.2 Measures

#### 2.2.1 Parenting stress

Parenting stress was measured by the Chinese version of the Parenting Stress Index-Short Form (PSI-SF) (11), which consists of 36 items and includes three dimensions: parenting distress (e.g., “Since having this child, I have hardly been able to do things I enjoy”), dysfunctional parent-child interaction (e.g., “My child rarely does things that make me feel happy”), and difficult child (e.g., “My child seems to cry and fuss more than other children”). Each item was rated on a 5-point scale ranging from 1 (strongly disagree) to 5 (strongly agree), with higher scores indicating greater subjective parenting stress. Confirmatory factor analysis indicated that the factor structure of the PSI-SF had a good fit to the data (RMSEA = 0.080, CFI = 0.939, TLI = 0.935, SRMR = 0.050). The scale also demonstrated excellent internal consistency (Cronbach’s α = 0.966).

#### 2.2.2 Harsh discipline

Harsh discipline was assessed by the Psychological Aggression (e.g., “Yell at your child angrily”), Corporal Punishment (e.g., “Spanking a child”), and Physical Abuse (e.g., “Throw the child forcefully to the ground or knock him/her down”) subscales of the Parent-Child Conflict Tactics Scale (CTSPC) (15), which consists of 14 items. The question presented in the questionnaire is “Please select the appropriate option based on the number of times you have engaged in the following behaviors with your child in the past month.” Each item was rated on a 6-point scale, ranging from 0 (never), 1 (once), 2 (twice), 3 (3–5 times), 4 (6–10 times), and 5 (10 times or more). The scale was scored based on the median occurrences of harsh discipline. This method follows the original scoring recommendation (15) and is commonly used to address the skewed distribution typical of such behaviors, providing a more robust indicator of an individual’s relative position within the sample and reducing the influence of extreme values (16). Higher scores indicate a more frequent occurrence of these behaviors. Confirmatory factor analysis indicated that the factor structure of the CTSPC had a good fit to the data (RMSEA = 0.078, CFI = 0.984, TLI = 0.981, SRMR = 0.032). The scale also demonstrated excellent internal consistency (Cronbach’s α = 0.922).

#### 2.2.3 Internalizing problems in children and adolescents

Internalizing problems in children and adolescents was measured by the internalizing subscale of the parent version of the Strength and Difficulty Questionnaire (SDQ) (36), which consists of 10 items and divides into two dimensions: emotional symptoms (e.g., “Frequently angry and irritable”) and peer interaction problems (e.g., “At least one good friend”). Each item was rated on a 3-point scale ranging from 0 (not applicable) to 2 (completely applicable), with higher scores indicating greater severity of internalizing problems. The Cronbach’s α for the scale in this study was 0.70. Confirmatory factor analysis indicated that the factor structure of the SDQ internalizing subscale had an excellent fit to the data (RMSEA = 0.048, CFI = 0.983, TLI = 0.978, SRMR = 0.040). The scale demonstrated acceptable internal consistency (Cronbach’s α = 0.703).

#### 2.2.4 Emotion regulation

The Emotion Regulation Questionnaire (ERQ) (37) was used to measure cognitive reappraisal (6 items, e.g., “When I want to feel positive emotions (such as happiness or joy), I change the way I think about things”) and expressive suppression (4 items, e.g., “When I feel positive emotions, I am very careful not to let them show”). Each item was rated on a 7-point scale ranging from 1 (strongly disagree) to 7 (strongly agree), with higher scores indicating more frequent use of the respective emotion regulation strategy. Confirmatory factor analysis indicated that the factor structure of the ERQ had a good fit to the data (RMSEA = 0.067, CFI = 0.986, TLI = 0.983, SRMR = 0.024). The subscales demonstrated good internal consistency (cognitive reappraisal: α = 0.885; expressive suppression: α = 0.843), and the total scale also demonstrated good reliability (Cronbach’s α = 0.867).

Acceptance was measured using the Acceptance and Action Questionnaire (AAQ) (38), which focuses on an individual’s degree of experiential avoidance across 7 items (e.g., “Painful memories have ruined my happy life”). Each item was rated on a 7-point scale ranging from 1 (never) to 7 (always), with higher scores indicating greater levels of experiential avoidance and lower acceptance. In this study, the questionnaire was reverse-scored to calculate an acceptance score, with higher scores indicating higher levels of acceptance. Confirmatory factor analysis indicated that the factor structure of the AAQ had a good fit to the data (RMSEA = 0.081, CFI = 0.996, TLI = 0.994, SRMR = 0.012). The scale demonstrated excellent internal consistency (Cronbach’s α = 0.939).

Rumination was measured by the Ruminative Responses Scale (RRS) (39), which consists of 10 items and includes two main subscales: forced meditation (e.g., “I often analyze my personality to understand why I feel depressed”) and deep reflection (e.g., “I often wonder what I did to cause this”). Each item was rated on a 4-point scale ranging from 1 (never) to 4 (always), with higher scores indicating greater levels of rumination. Confirmatory factor analysis indicated that the factor structure of the RRS had a good fit to the data (RMSEA = 0.087, CFI = 0.976, TLI = 0.970, SRMR = 0.034). The scale demonstrated excellent internal consistency (Cronbach’s α = 0.904).

Distraction was measured by the distraction subscale of the Behavioral Emotion Regulation Questionnaire (BERQ) (40), which consists of 4 items (e.g., “I threw myself into other unrelated activities”). Each item was rated on a 5-point scale, ranging from 1 (never) to 5(always), with higher scores indicating more frequent use of the distraction strategy during stressful events. The Cronbach’s α of the subscale in this study was 0.76. Confirmatory factor analysis indicated that the factor structure of the BERQ distraction subscale had a good fit to the data (RMSEA = 0.091, CFI = 0.994, TLI = 0.982, SRMR = 0.015). The subscale demonstrated acceptable internal consistency (Cronbach’s α = 0.758).

### 2.3 Data analysis

All analyses were performed using SPSS 23. First, Harman’s single-factor test was used to identify common method variance. Second, descriptive statistics and Pearson correlation analysis were conducted to examine the relationships among key variables and obtain further information. Third, mediation analysis was performed using the PROCESS macro v4.2 Model 4 to test the mediating role of harsh discipline in the relationship between parenting stress and internalizing problems in children and adolescents. Subsequently, Model 7 was employed to examine the moderating effect of emotion regulation on the relationship between parenting stress and harsh discipline (41). All significance tests used bias-corrected bootstrapping with 5000 resamples to generate 95% confidence interval (CI). Effects were considered statistically significant when the 95% CI excluded zero, indicating robust mediation effects.

### 2.4 Results

#### 2.4.1 Common method bias test

To mitigate potential common method bias, standardized procedures were followed during survey administration. Additionally, Harman’s single-factor test was conducted using principal component analysis. The results revealed 16 factors with initial eigenvalues greater than 1, and the first factor accounted for only 31.78% of the variance, below the critical threshold of 40% (42). The test showed that there was no obvious common method variance in this study.

#### 2.4.2 Descriptive statistics and correlation analysis

The means, standard deviations, and bivariate correlations are presented in **Table 1**. As shown, parenting stress was positively correlated with harsh discipline (*r* = 0.43, *p* < 0.01) and internalizing problems in children and adolescents (*r* = 0.58, *p* < 0.01). Similarly, harsh discipline was positively correlated with internalizing problems (*r* = 0.51, *p* < 0.01). These results indicate that the main variables in the proposed mediation model are positively correlated with each other.

**Table 1:** Descriptive statistics and correlations among the study 1 variables. (*N* = 971).

Variables	*M*	*SD*	1	2	3	4	5	6	7	8
Stress	91.98	29.79	1							
Discipline	20.52	30.16	0.43**	1						
Problem	5.92	3.48	0.58**	0.51**	1					
Reappraisal	31.04	6.52	0.01	-0.08*	-0.11**	1				
Suppression	17.56	5.53	0.30**	0.11**	0.28**	0.40**	1			
Rumination	24.67	6.31	0.41**	0.29**	0.41**	0.23**	0.37**	1		
Acceptance	31.91	10.62	-0.67**	-0.43**	-0.59**	-0.02	-0.30**	-0.61**	1	
Distraction	13.14	3.11	0.27**	0.17**	0.22**	0.33**	0.24**	0.51**	-0.46**	1

**p < 0.01. Stress, parenting stress; Discipline, harsh discipline; Problem, internalizing problems in children and adolescents.

#### 2.4.3 Mediation model test

**Figure 1:**
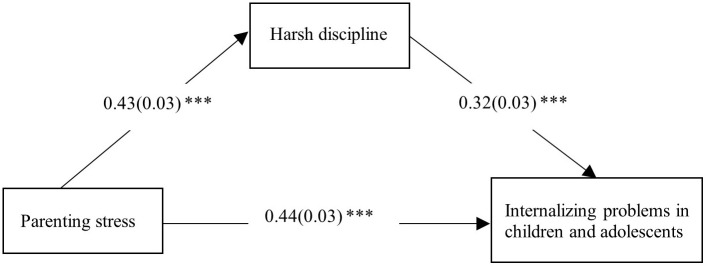
The mediating effect of harsh discipline in the association between parenting stress and internalizing problems in children and adolescents.*** p < 0.001.

Model 4 in the PROCESS macro for SPSS was used to test the mediation model. All variables were standardized. The results are shown in **Table 2**, **Figure 1**. Parenting stress was positively correlated with both harsh discipline (*β* = 0.43, *SE* = 0.03, *p* < 0.001) and internalizing problems in children and adolescents (*β* = 0.44, *SE* = 0.03, *p* < 0.001). Harsh discipline and internalizing problems were also positively correlated (*β* = 0.32, *SE* = 0.03, *p* < 0.001). The total effect of parenting stress on internalizing problems was significant (*total effect* = 0.58, *SE* = 0.03, 95% CI [0.52, 0.63]). The indirect effect of parenting stress on internalizing problems through harsh discipline was significant (*indirect effect* = 0.14, *SE* = 0.02, 95% CI [0.11, 0.17]), accounting for 24.14% of the total effect. After controlling for harsh discipline, the direct effect of parenting stress on internalizing problems remained significant (*direct effect* = 0.44, *SE* = 0.03, 95% CI [0.39, 0.50]). These results suggest that harsh discipline partially mediates the relationship between parenting stress and internalizing problems in children and adolescents.

**Table 2:** The mediation model in study 1.

Predictor variable	Outcome variable	R	R²	*F*	*β*	*t*	Boot LLCI	Boot ULCI
Stress	Discipline	0.43	0.19	221.20***	0.43	14.87***	0.37	0.49
Stress	Problem	0.65	0.42	351.64***	0.44	16.31***	0.39	0.50
Discipline					0.32	11.84***	0.27	0.37
Stress	Problem	0.58	0.34	492.29***	0.58	22.19***	0.53	0.63

***p < 0.001. Stress, parenting stress; Discipline, harsh discipline; Problem, internalizing problems in children and adolescents.

#### 2.4.4 Moderated mediation model test

Model 7 in the PROCESS macro for SPSS was used to test the moderated mediation model. All variables were standardized. The interaction effects between parenting stress and acceptance (*β* = -0.10, *SE* = 0.03, *p* < 0.001), distraction (*β* = 0.05, *SE* = 0.03, *p* < 0.05) and rumination (*β* = 0.09, *SE* = 0.03, *p* < 0.001) were statistically significant. The interaction effect between parenting stress and reappraisal (*β* = -0.05, *SE* = 0.03, *p* = 0.05) was marginally significant, while the interaction between parenting stress and suppression (*β* = 0.02, *SE* = 0.03, *p* = 0.58) was not significant. These results indicate that acceptance and reappraisal may reduce the effect of parenting stress on harsh discipline, whereas distraction and rumination may enhance it. Suppression did not moderate the relationship between parenting stress and harsh discipline.

**Figure 2:**
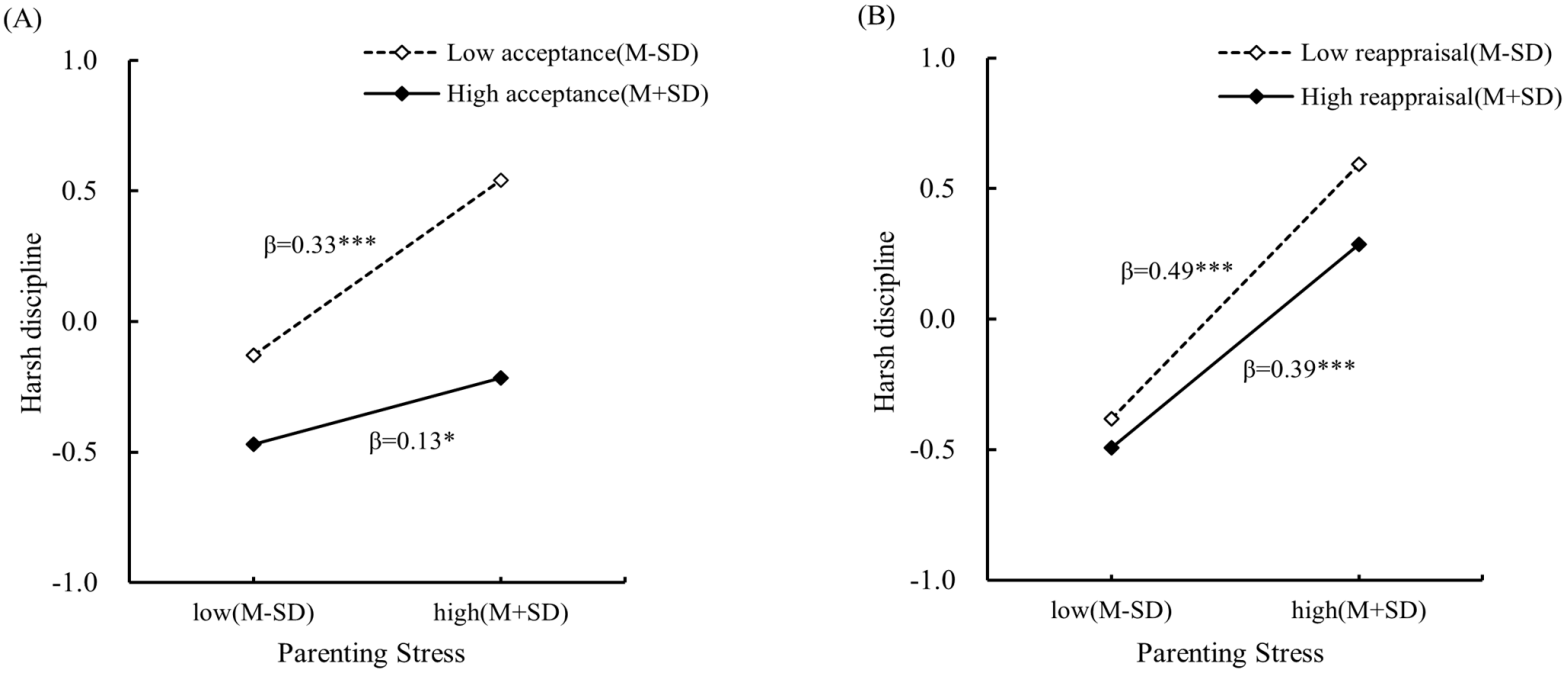
Moderating effect of **(A)** acceptance and **(B)** reappraisal on the relationship between parenting stress and harsh discipline. * p < 0.05, *** p < 0.001.

**Figure 3:**
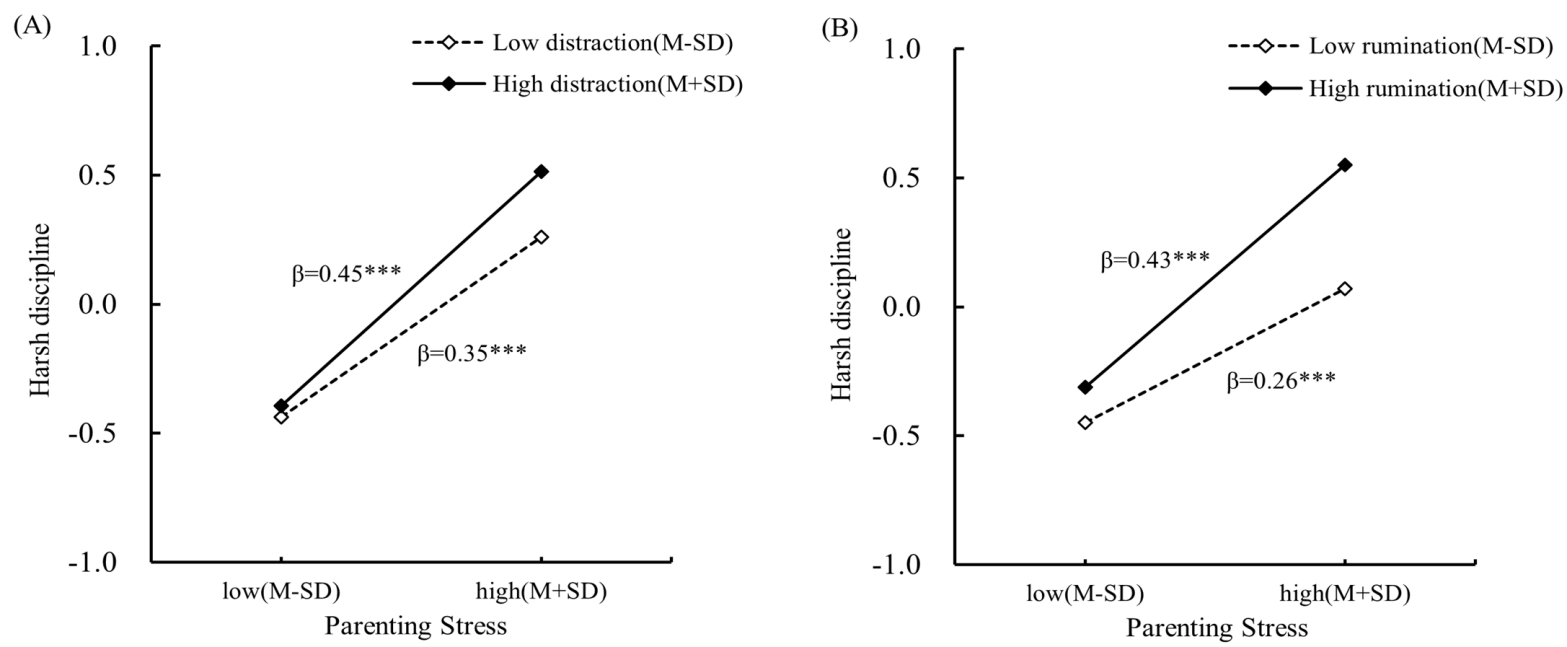
Moderating effect of **(A)** distraction and **(B)** rumination on the relationship between parenting stress and harsh discipline.*** p < 0.001.

To further interpret the interaction patterns, simple slope tests were conducted. As shown in **Figures 2**, **3**, the association between parenting stress on harsh discipline was weaker when individuals reported high acceptance or reappraisal (one standard deviation above the sample mean, acceptance: *βsimple* = 0.13, *t* = 2.54, *p* < 0.05; reappraisal: *βsimple* = 0.39, *t* = 10.84, *p* < 0.001) than when they reported low acceptance or reappraisal (one standard deviation below the sample mean, acceptance: *βsimple* = 0.33, *t* = 7.92, *p* < 0.001; reappraisal: *βsimple* = 0.49, *t* = 12.04, *p* < 0.001). Conversely, the association between parenting stress and harsh discipline was weaker when individuals reported low distraction or rumination (distraction: *βsimple* = 0.35, *t* = 7.94, *p* < 0.001; rumination: *βsimple* = 0.26, *t* = 5.53, *p* < 0.001) than when they reported high distraction or rumination (distraction: *βsimple* = 0.45, *t* = 12.97, *p* < 0.001; rumination: *βsimple* = 0.43, *t* = 12.31, *p* < 0.001).

## 3 Study 2

### 3.1 Participants

Participants were recruited from a school in Sichuan Province, China. All parents were invited via the school’s official communication channels to participate in a three-week online parenting training program. Inclusion criteria were identical to those in Study 1, with the following additions: (a) willingness to participate in the three-week online intervention courses and complete both pre-test and post-test assessments. Exclusion criteria were identical to those in Study 1, with the following additions: (a) for adolescents aged 10 and above, cognitive impairments or reading difficulties preventing self-report comprehension; (b) family participation in any structured parenting intervention or psychological treatment within the past three months.

Study 2 recruited 140 parents from a school in Sichuan who volunteered to participate in the intervention. After accounting for attrition, 123 parents completed the study, comprising 63 in the intervention group (89% female; *Mage* = 39.85, *SD* = 5.14) and 60 in the control group (40.9% female; *Mage* = 32.82, *SD* = 5.48). The majority (90.7%) resided in urban areas. In the intervention group, 47.6% had one child and 52.4% had two or more; 15.9% held qualifications below a bachelor’s degree, and 84.1% had a bachelor’s degree or higher. Family structures included two-parent households (55.4%), single-parent households (6.8%), and other arrangements (37.8%, e.g., living with grandparents or relatives). Grandparental involvement in child-rearing was reported by 42.9%. In the control group, 78.3% had one child and 21.7% had two or more; 20.0% held below-bachelor’s qualifications, and 80.0% had a bachelor’s degree or higher. Family structures consisted of two-parent households (62.1%) and other arrangements (37.9%). Grandparental involvement was reported by 65.0%.

### 3.2 Procedure

#### 3.2.1 Theoretical basis of intervention training courses

Study 1 demonstrates that acceptance and reappraisal buffer the negative impact of parenting stress on harsh discipline, identifying these strategies as protective factors that may disrupt the pathway from stress to maladaptive parenting. This finding aligns with broader evidence: mindfulness-based interventions emphasizing acceptance improve family functioning (43); reappraisal enhances parenting efficacy (44); and when combined with emotional empathy training, it improves parent-child interactions and reduces child behavioral problems. Together, these studies suggest that enhancing parental emotion regulation, particularly acceptance and reappraisal, can cultivate a healthier family emotional environment. Based on these findings, Study 2 designed an intervention training course focusing on emotion regulation to promote acceptance and reappraisal and foster positive parenting skills.

To reinforce the acquisition and daily application of these strategies, the intervention incorporated psychological diaries. Psychological diaries involve regularly recording thoughts, experiences, and feelings, allowing individuals to express reflections and emotions without reservation. As an auxiliary tool for emotion regulation training, this approach serves to release negative emotions and experiences (45). Specifically, it helps parents better apply acceptance and reappraisal to manage their emotions in parenting contexts. Over time, diary writing can strengthen individuals’ confidence in emotion regulation, enhance self-efficacy, and improve their ability to manage life stressors (46).

#### 3.2.2 Intervention training courses

The three-week intervention consisted of four core modules focusing on emotion regulation, parent-child interaction, child development, and parenting styles. Each module was built on the previous one to foster progressive development of emotion regulation skills from the intrapersonal to the interpersonal and systemic levels. An overview of the modules, content, regulatory targets, and course examples is provided in **Supplementary Table 1**.

The intervention was delivered online over 21 consecutive days. To maximize efficacy, it combined structured online courses with daily psychological diaries—a dual approach designed to reinforce learning, ensure engagement, and provide a space for emotional expression. The intervention group received daily links to course videos (approximately 45 minutes) via a WeChat group and were required to watch them and write psychological diaries. No specific format or length was required, but the diary focused on daily interactions with their children. Parents uploaded diaries to the WeChat “Check-in” mini-program, where teachers reviewed them and provided brief responses. This approach offered flexibility, while the daily diary submissions addressed the low adherence common in unsupervised online interventions. By fostering consistent involvement and providing individualized feedback, this approach enhanced engagement and helped reduce attrition rates. The primary aim of this intervention was to enhance parents’ acceptance and cognitive reappraisal abilities, thereby buffering the impact of parenting stress on harsh discipline and ultimately reducing child internalizing problems.

#### 3.2.3 Intervention procedure

In Study 2, participants were randomly assigned to an intervention group and a control group. Data were collected via the Wenjuanxing platform, with separate modules for parents and children to ensure clear and independent sources of information. At pretest, parents first signed an online informed consent form and then independently completed the demographic questionnaire, as well as the PSI-SF, CTSPC, ERQ, and AAQ. For children under 10 years of age, the same parent proceeded to complete the Rutter Child Behavior Questionnaire (Rutter) immediately after finishing their own module. For adolescents aged 10 and above, parents provided informed consent for their child’s participation. Subsequently, the researcher provided the adolescent with separate online links to the Screen for Child Anxiety Related Emotional Disorders (SCARED) and the Childhood Depression Inventory (CDI), instructing them to complete these measures in a quiet, private setting without parental presence or interference.

Following the pretest, the intervention group participated in the three-week program described in Section 3.2.2, while the control group maintained their regular work and life routines. During the intervention period, the intervention group received daily course links via a WeChat group, watched the videos, and submitted psychological diaries. Teachers reviewed diaries and provided brief responses. After the three-week intervention, participants from both groups completed the same posttest questionnaires as the pretest.

### 3.3 Measures

#### 3.3.1 Measurement instruments: selection rationale and comparability

The core variable across both studies was child internalizing problems. Study 1 employed the SDQ internalizing subscale, a widely used screening tool with good reliability and validity for preliminary large-scale assessment (36). Study 2 adopted a developmentally sensitive, multi-informant strategy. For children under 10, parental report remains the primary reliable source (6); the Rutter Child Behavior Questionnaire (parent version) was therefore used. For adolescents aged 10 and above, the SCARED and CDI were employed (8). This approach reduces single-informant bias and enhances construct validity, as self-report uniquely captures internalized emotional experiences (47).

To ensure cross-study comparability, construct consistency and score equivalence was addressed. Construct continuity was supported by high correlations between the Rutter and SDQ, reflecting the latter’s theoretical origins in the former (36, 48). The SCARED and CDI combination correlates strongly with the SDQ internalizing subscale and covers the anxiety and depression dimensions central to internalizing problems (49). To enable statistical comparability, raw scores from the Rutter and SCARED+CDI were standardized within the sample (M = 0, SD = 1), a widely used strategy for integrating data from heterogeneous measures in developmental psychopathology research (50). All other variables were measured with identical instruments across both studies, ensuring full measurement consistency.

#### 3.3.2 Measurement instruments

Parenting stress, harsh discipline, reappraisal, and acceptance were assessed using the same measures as in Study 1. Parenting stress was measured by the Chinese version of the Parenting Stress Index-Short Form (PSI-SF) (11). Confirmatory factor analysis indicated that the factor structure of the PSI-SF in Study 2 had a good fit to the data (RMSEA = 0.078, CFI = 0.947, TLI = 0.944, SRMR = 0.075). The scale demonstrated excellent internal consistency (Cronbach’s α = 0.966).Harsh discipline was assessed by the Psychological Aggression, Corporal Punishment, and Physical Abuse subscales of the Parent-Child Conflict Tactics Scale (CTSPC) (15). Confirmatory factor analysis indicated that the factor structure of the CTSPC in Study 2 had a good fit to the data (RMSEA = 0.080, CFI = 0.979, TLI = 0.974, SRMR = 0.055). The scale demonstrated excellent internal consistency (Cronbach’s α = 0.923).The Emotion Regulation Questionnaire (ERQ) (37) was used to measure reappraisal (6 items). Confirmatory factor analysis indicated that the factor structure of the ERQ reappraisal subscale in Study 2 had a good fit to the data (RMSEA = 0.095, CFI = 0.978, TLI = 0.973, SRMR = 0.048). The subscale demonstrated good internal consistency (Cronbach’s α = 0.827). Acceptance was measured using the Acceptance and Action Questionnaire (AAQ) (38). Confirmatory factor analysis indicated that the factor structure of the AAQ in Study 2 had an excellent fit to the data (RMSEA = 0.063, CFI = 0.995, TLI = 0.994, SRMR = 0.022). The scale demonstrated excellent internal consistency (Cronbach’s α = 0.897).

Internalizing problems in children under the age of 10 were measured by the Rutter Child Behavior Questionnaire Parent Questionnaire (51), which consists of 31 items and divides behavioral problems into two major categories: antisocial behavior (e.g., frequently destroying one’s own and others’ belongings) and neurotic behavior (e.g., stomachaches and vomiting). Each item was rated on a 3-point scale ranging from 0 (this situation has never occurred) to 2 (severe symptoms or frequent occurrence, or at least once a week), with higher scores indicating greater severity of internalizing problems. Confirmatory factor analysis indicated that the factor structure of the Rutter scale had a good fit to the data (RMSEA = 0.049, CFI = 0.975, TLI = 0.973, SRMR = 0.118). The scale demonstrated excellent internal consistency (Cronbach’s α = 0.954).

Internalizing problems in children aged 10 and older were measured by the Screen for Child Anxiety Related Emotional Disorders(SCARED) (52) and the Childhood Depression Inventory (CDI) (53). This study selected two subscales from the SCARED: social phobia (e.g., feeling tense when with unfamiliar people) and school phobia (e.g., feeling headaches at school), totaling 11 items. Each item was rated on a 3-point scale ranging from 1 (none) to 3 (often), with higher scores indicating more severe anxiety. Confirmatory factor analysis indicated that the factor structure of the SCARED had an excellent fit to the data (RMSEA = 0.012, CFI = 1.000, TLI = 1.000, SRMR = 0.059). The combined scale demonstrated excellent internal consistency (Cronbach’s α = 0.923). The CDI consists of 27 items and is divided into five dimensions: anhedonia, negative mood, negative self-esteem, ineffectiveness, and interpersonal problems. Each item consists of three sentences with the same structure, and respondents must select the option that best describes their feelings (e.g., “I occasionally feel unhappy,” “I often feel unhappy,” “I always feel unhappy”), with scores ranging from 0 to 2. A higher total score indicates more severe depression. The Cronbach’s α of the scale in this study was 0.75. Confirmatory factor analysis indicated that the factor structure of the CDI had a good fit to the data (RMSEA = 0.063, CFI = 0.945, TLI = 0.939, SRMR = 0.128). The scale demonstrated acceptable internal consistency (Cronbach’s α = 0.755).

### 3.4 Data analysis

All analyses were conducted using SPSS 23. First, Harman’s single-factor test was used to identify common method variance. Second, descriptive statistics and Pearson correlation analyses were performed to examine relationships among key variables. Third, Study 2 tested whether harsh discipline mediated the association between parenting stress and internalizing problems in children and adolescents, replicating Study 1 in an independent sample. Mediation analysis was conducted using the PROCESS macro v4.2 Model 4. Next, an independent samples t-test compared pre-test results between the intervention and control groups to confirm baseline equality. Finally, a two-way repeated measures analysis of variance (ANOVA) was applied to evaluate the changes of emotion regulation in post intervention training courses and intervention effects on parenting stress, harsh discipline and internalizing problems.

### 3.5 Results

#### 3.5.1 Common method bias test

To mitigate potential common method bias, standardized procedures were followed during survey administration. Additionally, Harman’s single-factor test was conducted using principal component analysis. The results revealed 17 factors with initial eigenvalues greater than 1, and the first factor accounted for only 30.11% of the variance, below the critical threshold of 40% (42). The test showed that there was no obvious common method variance in this study.

#### 3.5.2 Descriptive statistics and correlation analysis of pre-test

The means, standard deviations, and bivariate correlations of pre-test results are presented in **Table 3**. To ensure cross-age comparability, all scores from several age-appropriate questionnaires assessing internalizing problems were standardized by converting them to z-scores using the data of each measure. As shown, parenting stress was positively correlated with harsh discipline (*r* = 0.55, *p*<0.01) and internalizing problems in children and adolescents (*r* = 0.64, *p*<0.01). Similarly, harsh discipline was positively correlated with internalizing problems (*r* = 0.52, *p* < 0.01). These results indicate that, in the pre-test, the main variables in the proposed mediation model are positively correlated with each other.

**Table 3:** Descriptive statistics and correlations among the study 2 variables of pre-test results. (*N* = 123).

Variables	*M*	*SD*	1	2	3
Stress	86.10	25.09	1		
Discipline	13.09	12.00	0.55**	1	
Problem	0.00	1.00	0.64**	0.52**	1

**p < 0.01. Stress, parenting stress; Discipline, harsh discipline; Problem, internalizing problems in children and adolescents.

#### 3.5.3 Mediation model test of pre-test

**Figure 4:**
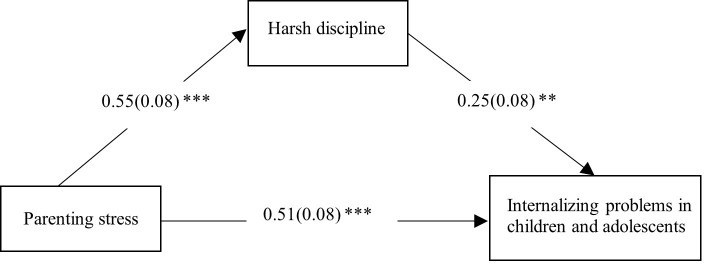
The mediating effect of harsh discipline in the association between parenting stress and internalizing problems in children and adolescents in the pre-test.** p < 0.01, *** p < 0.001.

Model 4 in the PROCESS macro for SPSS was used to test the mediation model of pre-test results. All variables were standardized. The results are shown in **Table 4**, **Figure 4**. Parenting stress was positively correlated with both harsh discipline (*β* = 0.55, *SE* = 0.08, *p* < 0.001) and internalizing problems in children and adolescents (*β* = 0.51, *SE* = 0.08, *p* < 0.001). Harsh discipline and internalizing problems were also positively correlated (*β* = 0.25, *SE* = 0.08, *p* < 0.01). The total effect of parenting stress on internalizing problems was significant (*total effect* = 0.64, *SE* = 0.07, 95% CI [0.50, 0.78]). The indirect effect of parenting stress on internalizing problems through harsh discipline was significant (*indirect effect* = 0.13, *SE* = 0.04, 95% CI [0.06, 0.22]), accounting for 20.31% of the total effect. After controlling for harsh discipline, the direct effect of parenting stress on internalizing problems remained significant (*direct effect* = 0.51, *SE* = 0.08, 95% CI [0.35, 0.66]). These results suggest that, in the pre-test, harsh discipline partially mediates the relationship between parenting stress and internalizing problems in children and adolescents.

**Table 4:** The mediation model in study 2 pre-test.

Predictor variable	Outcome variable	R	R²	*F*	*β*	*t*	Boot LLCI	Boot ULCI
Stress	Discipline	0.55	0.30	51.12***	0.55	7.15***	0.39	0.70
Stress	Problem	0.68	0.46	50.17***	0.51	6.32***	0.35	0.66
Discipline					0.25	3.07**	0.09	0.40
Stress	Problem	0.64	0.41	85.01***	0.64	9.22***	0.50	0.78

**p < 0.01, ***p < 0.001. Stress, parenting stress; Discipline, harsh discipline; Problem, internalizing problems in children and adolescents.

#### 3.5.4 Pre-test difference test between the intervention and control groups.

An independent samples t-test was conducted on pre-test scores to assess baseline equality between the intervention and control groups. Internalizing problem scores were standardized to account for different measurement scales. As shown in **Table 5**, no significant differences were observed in any baseline variables, confirming group homogeneity before the intervention.

**Table 5:** Tests for pre-test differences in study 2 variables.

Variables	Group(*M* ± *SD*)		*t*	*p*	*Cohen’s d*
	Intervention group	Control group			
Stress	83.63 ± 20.35	88.68 ± 29.22	-1.12	0.27	-0.20
Discipline	12.63 ± 11.96	13.57 ± 12.13	-0.43	0.67	-0.08
Problem	-0.07 ± 0.88	0.07 ± 1.11	-0.76	0.45	-0.14
Acceptance	36.48 ± 6.82	35.67 ± 8.71	0.58	0.57	0.11
reappraisal	31.25 ± 5.75	32.93 ± 4.31	-1.83	0.07	0.33

**p < 0.01, ***p < 0.001. Stress, parenting stress; Discipline, harsh discipline; Problem, internalizing problems in children and adolescents.

#### 3.5.5 Manipulation check: changes of emotion regulation in post intervention training courses

**Figure 5:**
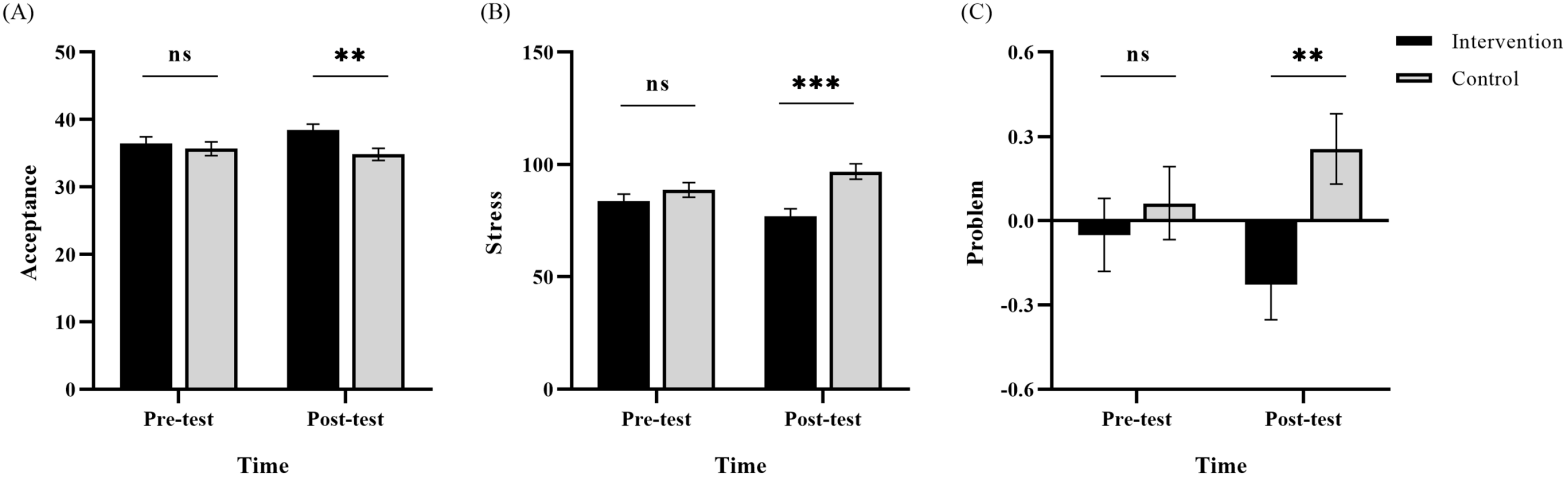
The time and group interaction for **(A)** acceptance, **(B)** parenting stress and **(C)** internalizing problem. ns p > 0.05, ** p < 0.01, *** p < 0.001.

A two-way repeated measures ANOVA was conducted to test for the changes of emotion regulation in post intervention training courses between the two groups. For acceptance, as shown in **Figure 5A**, the interaction between time (pre-test vs. post-test) and group (intervention vs. control) was significant, *F* (1, 121) = 5.00, *p* < 0.05, *η_p_²* = 0.04. Further simple effect analysis indicated that at the post-test, acceptance in the intervention group (*M* = 38.43, S*D* = 5.77) was significantly higher than that in the control group (*M* = 36.48, *SD* = 6.82) (*p* < 0.01). However, the interaction of reappraisal was not significant, *F* (1, 121) = 0.01, *p* > 0.05, *η_p_²* = 0.00. These findings collectively indicate that the intervention training courses have an effective effect on increasing acceptance but no effect on reappraisal.

#### 3.5.6 Intervention effects on parenting stress, harsh discipline and internalizing problems

A two-way repeated measures ANOVA was conducted to test for differences between the two groups in terms of parenting stress, harsh discipline and internalizing problems in children and adolescents. For parenting stress, as shown in **Figure 5B**, the interaction between time (pre-test vs. post-test) and group (intervention vs. control) was significant, *F* (1, 121) = 10.16, *p* < 0.01, *η_p_*² = 0.08. Further simple effect analysis indicated that at the post-test, parenting stress in the intervention group (*M* = 76.94, *SD* = 3.36) was significantly lower than that in the control group (*M* = 96.85, *SD* = 3.45) (*p* < 0.001). Similarly, as shown in **Figure 5C**, standardized internalizing problem scores demonstrated a significant interaction, *F* (1,121) = 5.36, *p* = .023, *ηp²* = 0.04. Further simple effect analysis indicated that at the post-test, internalizing problems in the intervention group (*M* = -0.26, *SD* = 0.12) were significantly lower than those in the control group (*M* = 0.27, *SD* = 0.12) (*p* < 0.01).

The main effect of time on harsh discipline was significant, *F* (1,121) = 6.51, *p* < 0.05, *η_p_*² = 0.05, with post-test scores (*M* = 10.57, *SD* = 0.77) significantly lower than pre-test scores (*M* = 13.10, *SD* = 1.09). The time and group interaction was not significant, *F* (1,121) =1.93, *p* = 0.167, *η_p_*² = 0.02. Although the interaction did not reach statistical significance, further analysis based on research hypotheses and pre-planned procedures revealed: At pre-test, no significant difference existed between the intervention group (*M* = 12.64, *SD* = 1.52) and control group (*M* = 13.57, *SD* = 1.56) (*p* > 0.05); At post-test, the intervention group (*M* = 8.73, *SD* = 1.08) were significantly lower than the control group (*M* = 12.42, *SD* = 1.10)(*p* < 0.05). This pattern aligns with the expected direction of the intervention effect, providing preliminary support for the intervention’s potential influence on harsh discipline. These findings collectively indicate the intervention’s robust effects on reducing parenting stress and internalizing problems in children and adolescents, with more tentative evidence for impacts on harsh discipline.

## 4 Discussion

Consistent with the Family Stress Model (12), Study 1 found that parenting stress was positively associated with child internalizing problems, and this relationship was partially mediated by harsh discipline. This pattern aligns with the theoretical proposition that stressors permeate the family system by impairing parenting behaviors. Specifically, parenting stress may trigger negative emotional reactions and deplete cognitive resources necessary for constructive parenting, thereby increasing the likelihood of harsh disciplinary responses (54). Once enacted, harsh discipline, whether psychological aggression or corporal punishment, has been consistently linked to elevated internalizing symptoms in children (17, 55). The observed partial mediation suggests that while harsh discipline represents a critical pathway, other mechanisms, such as parental mental health or parent–child attachment, likely also contribute. These findings extend the Family Stress Model by identifying harsh discipline as a specific behavioral mechanism linking parenting stress to child internalizing problems and provide a foundation for targeting parenting behaviors in interventions.

Study 1 further revealed that the link between parenting stress and harsh discipline was differentially moderated by specific emotion regulation strategies. The buffering effects of acceptance and reappraisal are consistent with the process model of emotion regulation (23, 31), which posits that antecedent-focused strategies modify the emotional trajectory before response tendencies become fully activated. Acceptance may enable parents to acknowledge stressful emotions without judgment, reducing experiential avoidance that often escalates into explosive reactions (56). Cognitive reappraisal may be associated with lower levels of harsh discipline by reframing child misbehavior (e.g., interpreting a tantrum as distress rather than defiance), thereby diminishing hostile attributions that trigger punitive responses (30). Both strategies, by targeting emotional experience early, may help preserve the cognitive resources needed for constructive parenting. In contrast, both distraction and rumination exacerbated the association between parenting stress and harsh discipline Although these strategies differ in form, with distraction representing attentional disengagement and rumination representing perseverative cognitive focus, they share a common limitation: both are passive strategies that fail to address the root causes of parenting stress (31). Distraction may temporarily divert attention from stressful situations but does not resolve underlying stressors; once distraction ceases, unresolved stress may rebound with intensified negative emotions (57). Rumination may amplify negative affect through repetitive focus on stress-related thoughts, potentially reinforcing hostile interpretations of child behavior (29). Thus, under high parenting stress, both strategies may heighten the risk of punitive disciplinary responses. These findings suggest that the adaptiveness of emotion regulation strategies is context-dependent: strategies that fail to address the source of stress may prove maladaptive under high parenting demands.

Expressive suppression did not moderate the relationship between parenting stress and harsh discipline. As a response-focused strategy, suppression modulates the outward expression of emotions without altering the underlying emotional experience (23). Under chronic parenting stress, prolonged use of suppression may deplete cognitive resources, impairing self-regulatory capacity and potentially increasing susceptibility to impulsive, harsh discipline (58). However, the null effect suggests that the consequences of suppression may depend on other factors, such as frequency of use or parenting context (31). Alternatively, suppression’s effects may accumulate over time rather than moderating the immediate stress–discipline relationship, a possibility that warrants longitudinal investigation.

Study 2 provided further evidence for the mediating role of harsh discipline in an independent sample, reinforcing the robustness of this mechanism and supporting the generalizability of the theoretical model across Chinese family contexts. Building on this replication, the three-week online intervention targeting emotion regulation yielded differential effects on specific regulatory strategies. The intervention significantly increased parents’ use of acceptance. This increase may reflect the intervention’s focus on nonjudgmental awareness and emotional tolerance, reinforced by daily psychological diaries that encouraged parents to openly acknowledge and reflect on their emotions (59). However, the intervention did not significantly increase cognitive reappraisal. This null effect may stem from the brief, standardized online format, which may have provided insufficient contextualized training and personalized feedback to support mastery of this complex cognitive skill (31). Alternatively, changes in reappraisal may require longer consolidation periods. They may manifest only after parents have had sustained opportunities to apply the strategy in real-world parenting situations, a possibility warranting follow-up assessment.

Despite no change in reappraisal, the intervention significantly reduced parenting stress and internalizing problems in children and adolescents. The increase in acceptance may have helped alleviate parents’ perceptions of parenting stress, consistent with findings from mindfulness-based parenting interventions (43). Daily diary writing may have further contributed to stress reduction by promoting emotional expression and reducing emotional avoidance (59). The improvement in child internalizing outcomes may operate through two pathways. First, enhanced parental emotion regulation, particularly acceptance, may improve family emotional climate and reduce parent–child conflict (60). Second, children may learn healthier emotion management by observing parents’ adaptive regulation (61).

The non-significant group-by-time interaction for harsh discipline warrants careful interpretation. Although the interaction did not reach significance, the intervention group showed significantly lower harsh discipline than the control group at post-test, with means in the hypothesized direction. The null interaction may be partially attributable to the intervention timing, which began during summer vacation, when reduced academic stress and increased parent–child time may have naturally lowered conflict in both groups, as reflected in the significant time effect. This shared trend may have attenuated the interaction despite meaningful group differences at post-test. Additionally, behavioral change often lags behind emotional and cognitive improvements; translating enhanced regulation into sustained disciplinary change may require extended consolidation beyond three weeks (62). Future interventions should consider longer training periods and follow-up assessments to capture delayed effects.

Several theoretical contributions emerge from this integrated approach. First, the research provides empirical support for the Family Stress Model (12) by demonstrating the mediating role of harsh discipline in the Chinese cultural context and its modifiability. Second, the findings are consistent with emotion regulation theory (23, 31) while revealing the context-dependent effectiveness of specific strategies: acceptance and reappraisal emerged as protective factors, whereas distraction and rumination were associated with exacerbated risk under high parenting stress. The null effect of suppression aligns with the limited resources model (63), suggesting that suppression may deplete cognitive resources under chronic stress. The protective role of acceptance was further supported by the intervention’s success in increasing acceptance and reducing parenting stress and child internalizing problems, suggesting that acceptance may be readily enhanced through brief online training. Practically, these findings support multi-level interventions. At the individual level, acceptance training may yield measurable benefits within a brief online format. At the family level, emotion regulation assessment could be integrated into routine child mental health services. At the community and policy levels, scalable online programs combining psychoeducation with reflective practice tools offer a potentially cost-effective approach to promoting family mental health.

## 5 Limitations and prospects

Several limitations should be acknowledged. First, the sample relied on single-parent reports, limiting generalizability. Future research could include more diverse samples. Second, the cross-sectional design of Study 1 precludes causal conclusions. Although the model posits emotion regulation as a moderator and harsh discipline as a mediator, observed associations may be bidirectional or influenced by unmeasured variables (e.g., child temperament). Future research could employ longitudinal designs or experimental manipulations to establish causal relationships. Third, potential moderators (e.g., child temperament, marital quality) and alternative mediators (e.g., parental mental health) were not examined. Fourth, internalizing problems were assessed using a developmentally sensitive but methodologically heterogeneous approach. Although statistical standardization enhanced cross-group comparability, informant biases remain. Future studies could incorporate multi-informant strategies (e.g., teacher reports, clinical interviews). Lastly, the 21-day intervention lacked long-term follow-up, limiting conclusions about sustained effects. The null effects on reappraisal and harsh discipline may reflect the brief format or delayed behavioral change. Future interventions could extend training duration and include long-term follow-ups (e.g., 3–6 months). Integrating dynamic monitoring and multidimensional assessments could further elucidate how parenting stress influences child development and inform more targeted family interventions.

## 6 Conclusion

Taken together, the two studies provide methodologically complementary support for the proposed moderated mediation model. Study 1 identified harsh discipline as a key mediator linking parenting stress to child internalizing problems and further provided evidence that emotion regulation strategies moderated this pathway, with acceptance and cognitive reappraisal functioning as protective buffers and distraction and rumination as potential risk factors. Study 2 experimentally targeted the protective strategies, providing evidence that an emotion regulation-focused intervention reduced parenting stress and child internalizing problems, with preliminary evidence of impact on harsh discipline. The convergence of findings across the two studies strengthens confidence in the robustness of these mechanisms. Together, this two-study approach offers a template for translating basic research into actionable intervention targets and highlight the promise of emotion regulation-centered strategies in promoting family-based mental health.

## Data Availability

The raw data supporting the conclusions of this article will be made available by the authors, without undue reservation.

## References

[1] Polanczyk GV Salum GA Sugaya LS Caye A Rohde LA . Annual research review: A meta‐analysis of the worldwide prevalence of mental disorders in children and adolescents. J Child Psychol Psychiatry. (2015) 56:345–65. doi: 10.1111/jcpp.12381. PMID: 25649325

[2] Willner CJ Gatzke-Kopp LM Bray BC . The dynamics of internalizing and externalizing comorbidity across the early school years. Dev Psychopathol. (2016) 28:1033–52. doi: 10.1017/S0954579416000687. PMID: 27739391 PMC5319409

[3] Muhtadie L Zhou Q Eisenberg N Wang Y . Predicting internalizing problems in Chinese children: The unique and interactive effects of parenting and child temperament. Dev Psychopathol. (2013) 25:653–67. doi: 10.1017/S0954579413000084. PMID: 23880383 PMC3725646

[4] Xu F Zhang L Wei X Zhang W Chen L Ji L . The stability of internalizing problem and its relation to maternal parenting during early adolescence. Psychol Dev Educ. (2015) 31:204–11. doi: 10.16187/j.cnki.issn1001-4918.2015.02.10

[5] Løhre A Lydersen S Vatten LJ . Factors associated with internalizing or somatic symptoms in a cross-sectional study of school children in grades 1-10. Child Adolesc Psychiatry Ment Health. (2010) 4:33. doi: 10.1186/1753-2000-4-33. PMID: 21167024 PMC3019130

[6] De Los Reyes A Augenstein TM Wang M Thomas SA Drabick DAG Burgers DE . The validity of the multi-informant approach to assessing child and adolescent mental health. Psychol Bull. (2015) 141:858–900. doi: 10.1037/a0038498. PMID: 25915035 PMC4486608

[7] Lee Y Kim B-N Park M-H Park S . Familial, cognitive, and behavioral characteristics of adolescents with depression. J Korean Acad Child Adolesc Psychiatry. (2017) 28:168–73. doi: 10.5765/jkacap.2017.28.3.168

[8] Jamnik MR DiLalla LF . Health outcomes associated with internalizing problems in early childhood and adolescence. Front Psychol. (2019) 10:60. doi: 10.3389/fpsyg.2019.00060. PMID: 30761037 PMC6362404

[9] Smetana JG Campione-Barr N Metzger A . Adolescent development in interpersonal and societal contexts. Annu Rev Psychol. (2006) 57:255–84. doi: 10.1146/annurev.psych.57.102904.190124. PMID: 16318596

[10] Bronfenbrenner U . The ecology of human development: Experiments by nature and design. Cambridge, MA: Harvard University Press (1979). doi: 10.4159/9780674028845

[11] Abidin R Flens J Austin W . The Parenting Stress Index. In Archer RP (Ed.), Forensic Uses Clin Assess Instrum. Lawrence Erlbaum Associates Publishers. (2006) 297–328. doi: 10.15347/wjm/2022.003

[12] Wu Q Xu Y . Parenting stress and risk of child maltreatment during the COVID-19 pandemic: A family stress theory-informed perspective. Dev Child Welf. (2020) 2:180–96. doi: 10.1177/2516103220967937. PMID: 41930703

[13] Tan TX Camras LA Deng H Zhang M Lu Z . Family stress, parenting styles, and behavioral adjustment in preschool-age adopted Chinese girls. Early Child Res Q. (2012) 27:128–36. doi: 10.1016/j.ecresq.2011.04.002. PMID: 41932720

[14] Erath SA El‐Sheikh M Mark Cummings E . Harsh parenting and child externalizing behavior: Skin conductance level reactivity as a moderator. Child Dev. (2009) 80:578–92. doi: 10.1111/j.1467-8624.2009.01280.x. PMID: 19467012 PMC2881831

[15] Straus MA Hamby SL Finkelhor D Moore DW Runyan D . Identification of child maltreatment with the Parent-Child Conflict Tactics Scales: Development and psychometric data for a national sample of American parents. Child Abuse Negl. (1998) 22:249–70. doi: 10.1016/S0145-2134(97)00174-9. PMID: 9589178

[16] Wang M Liu L . Parental harsh discipline in mainland China: Prevalence, frequency, and coexistence. Child Abuse Negl. (2014) 38:1128–37. doi: 10.1016/j.chiabu.2014.02.016. PMID: 24661692

[17] Lee HY Kim I Nam S Jeong J . Adverse childhood experiences and the associations with depression and anxiety in adolescents. Child Youth Serv Rev. (2020) 111:104850. doi: 10.1016/j.childyouth.2020.104850. PMID: 41932720

[18] Wang M Wang X Liu L . Paternal and maternal psychological and physical aggression and children’s anxiety in China. Child Abuse Negl. (2016) 51:12–20. doi: 10.1016/j.chiabu.2015.11.018. PMID: 26704300

[19] Rodriguez CM . Parental discipline and abuse potential affects on child depression, anxiety, and attributions. J Marriage Fam. (2003) 65:809–17. doi: 10.1111/j.1741-3737.2003.00809.x. PMID: 41933073

[20] Abidin RR . The determinants of parenting behavior. J Clin Child Psychol. (1992) 21:407–12. doi: 10.1207/s15374424jccp2104_12, PMID: 42045985

[21] Anthony LG Anthony BJ Glanville DN Naiman DQ Waanders C Shaffer S . The relationships between parenting stress, parenting behaviour and preschoolers’ social competence and behaviour problems in the classroom. Infant Child Dev. (2005) 14:133–54. doi: 10.1002/icd.385. PMID: 41925078

[22] Mikolajczak M Roskam I . A theoretical and clinical framework for parental burnout: The balance between risks and resources (BR2). Front Psychol. (2018) 9:886. doi: 10.3389/fpsyg.2018.00886. PMID: 29946278 PMC6006266

[23] Gross JJ . The emerging field of emotion regulation: An integrative review. Rev Gen Psychol. (1998) 2:271–99. doi: 10.1037/1089-2680.2.3.271. PMID: 41770175

[24] Mackler JS Kelleher RT Shanahan L Calkins SD Keane SP O’Brien M . Parenting stress, parental reactions, and externalizing behavior from ages 4 to 10. J Marriage Fam. (2015) 77:388–406. doi: 10.1111/jomf.12163. PMID: 26778852 PMC4712732

[25] Crandall A Deater-Deckard K Riley AW . Maternal emotion and cognitive control capacities and parenting: A conceptual framework. Dev Rev. (2015) 36:105–26. doi: 10.1016/j.dr.2015.01.004. PMID: 26028796 PMC4445866

[26] Liu L Cheng Q Zhu H . The impact of psychological resilienceon marital satisfaction: The chain mediating role of cognitive reappraisal and parenting stress. Adv Psychol. (2022) 12:308–16. doi: 10.12677/AP.2022.121035

[27] Mennin DS Ellard KK Fresco DM Gross JJ . United we stand: Emphasizing commonalities across cognitive-behavioral therapies. Behav Ther. (2013) 44:234–48. doi: 10.1016/j.beth.2013.02.004. PMID: 23611074 PMC4992341

[28] Nolen-Hoeksema S Morrow J . A prospective study of depression and posttraumatic stress symptoms after a natural disaster: The 1989 Loma Prieta earthquake. J Pers Soc Psychol. (1991) 61:115–21. doi: 10.1037/0022-3514.61.1.115. PMID: 1890582

[29] Thiruchselvam R Blechert J Sheppes G Rydstrom A Gross JJ . The temporal dynamics of emotion regulation: An EEG study of distraction and reappraisal. Biol Psychol. (2011) 87:84–92. doi: 10.1016/j.biopsycho.2011.02.009. PMID: 21354262

[30] Gross JJ . Emotion regulation: Affective, cognitive, and social consequences. Psychophysiology. (2002) 39:281–91. doi: 10.1017/S0048577201393198. PMID: 12212647

[31] Gross JJ . Emotion regulation: Current status and future prospects. Psychol Inq. (2015) 26:1–26. doi: 10.1080/1047840X.2014.940781. PMID: 41909888

[32] Prinz RJ . A population approach to parenting support and prevention: The Triple P System. Future Child. (2019) 29:122–43. doi: 10.1353/foc.2019.0005. PMID: 34409987

[33] Sullivan ADW Forehand R Acosta J Parent J Comer JS Loiselle R . COVID-19 and the acceleration of behavioral parent training telehealth: Current status and future directions. Cognit Behav Pract. (2021) 28:618–29. doi: 10.1016/j.cbpra.2021.06.012. PMID: 34629838 PMC8488182

[34] David OA Iuga IA Miron IS . Parenting: There is an app for that. A systematic review of parenting interventions apps. Child Youth Serv Rev. (2024) 156:107385. doi: 10.1016/j.childyouth.2023.107385. PMID: 41932720

[35] David OA Fodor LA Dascăl MD Miron IS . The efficacy of online parenting interventions in addressing emotional problems in children and adolescents: A meta-analysis of randomized controlled trials. Int J Soc Psychiatry. (2023) 69:1100–12. doi: 10.1177/00207640231156034. PMID: 36860086

[36] Goodman R . The Strengths and Difficulties Questionnaire: A research note. J Child Psychol Psychiatry. (1997) 38:581–6. doi: 10.1111/j.1469-7610.1997.tb01545.x. PMID: 9255702

[37] Gross JJ John OP . Individual differences in two emotion regulation processes: Implications for affect, relationships, and well-being. J Pers Soc Psychol. (2003) 85:348–62. doi: 10.1037/0022-3514.85.2.348. PMID: 12916575

[38] Bond FW Hayes SC Baer RA Carpenter KM Guenole N Orcutt HK . Preliminary psychometric properties of the Acceptance and Action Questionnaire–II: A revised measure of psychological inflexibility and experiential avoidance. Behav Ther. (2011) 42:676–88. doi: 10.1016/j.beth.2011.03.007. PMID: 22035996

[39] Treynor W Gonzalez R Nolen-Hoeksema S . Rumination reconsidered: A psychometric analysis. Cognit Ther Res. (2003) 27:247–59. doi: 10.1023/A:1023910315561. PMID: 41886696

[40] Kraaij V Garnefski N . The Behavioral Emotion Regulation Questionnaire: Development, psychometric properties and relationships with emotional problems and the Cognitive Emotion Regulation Questionnaire. Pers Individ Differ. (2019) 137:56–61. doi: 10.1016/j.paid.2018.07.036. PMID: 41932720

[41] Hayes A . Introduction to mediation, moderation, and conditional process analysis: A regression-based approach. Mediat Moderat Cond Process Anal. (2013) 1:12–20. doi: 10.1017/9781316995808.037. PMID: 41822556

[42] Podsakoff PM MacKenzie SB Lee J-Y Podsakoff NP . Common method biases in behavioral research: A critical review of the literature and recommended remedies. J Appl Psychol. (2003) 88:879–903. doi: 10.1037/0021-9010.88.5.879. PMID: 14516251

[43] Bögels SM Lehtonen A Restifo K . Mindful parenting in mental health care. Mindfulness. (2010) 1:107–20. doi: 10.1007/s12671-010-0014-5. PMID: 21125026 PMC2987569

[44] Hajal NJ Paley B . Parental emotion and emotion regulation: A critical target of study for research and intervention to promote child emotion socialization. Dev Psychol. (2020) 56:403–17. doi: 10.1037/dev0000864. PMID: 32077713

[45] Pennebaker JW Beall SK . Confronting a traumatic event: Toward an understanding of inhibition and disease. J Abnorm Psychol. (1986) 95:274–81. doi: 10.1037/0021-843X.95.3.274. PMID: 3745650

[46] Bandura A Caprara GV Barbaranelli C Pastorelli C Regalia C . Sociocognitive self-regulatory mechanisms governing transgressive behavior. J Pers Soc Psychol. (2001) 80:125–35. doi: 10.1037/0022-3514.80.1.125. PMID: 11195885

[47] Korelitz KE Garber J . Congruence of parents’ and children’s perceptions of parenting: A meta-analysis. J Youth Adolesc. (2016) 45:1973–95. doi: 10.1007/s10964-016-0524-0. PMID: 27380467 PMC5222679

[48] Van Roy B Veenstra M Clench‐Aas J . Construct validity of the five‐factor Strengths and Difficulties Questionnaire (SDQ) in pre‐, early, and late adolescence. J Child Psychol Psychiatry. (2008) 49:1304–12. doi: 10.1111/j.1469-7610.2008.01942.x. PMID: 19120709

[49] Kouros CD Wee SE Carson CN Ekas NV . Children’s self-blame appraisals about their mothers’ depressive symptoms and risk for internalizing symptoms. J Fam Psychol. (2020) 34:534–43. doi: 10.1037/fam0000639. PMID: 32027152 PMC7375012

[50] Griffith LE Van Den Heuvel E Raina P Fortier I Sohel N Hofer SM . Comparison of standardization methods for the harmonization of phenotype data: An application to cognitive measures. Am J Epidemiol. (2016) 184:770–8. doi: 10.1093/aje/kww098. PMID: 27769990 PMC5141949

[51] Goodman R . A modified version of the Rutter Parent Questionnaire including extra items on children’s strengths: A research note. J Child Psychol Psychiatry. (1994) 35:1483–94. doi: 10.1111/j.1469-7610.1994.tb01289.x. PMID: 7868642

[52] Wang K Su L Zhu Y Zhai J Yang Z Zhang J . Norms of the screen for child anxiety related emotional disorders in chinese urban children. Chin J Clin Psychol. (2002) 3:270–2. doi: 10.3969/j.issn.1005-3611.2002.04.009. PMID: 35900448

[53] Yu D Li X . Preliminary use of the children’s depression inventory in China. Chin Ment Health J. (2000) 14:225–7.

[54] Karreman A Van Tuijl C Van Aken MAG Deković M . Parenting, coparenting, and effortful control in preschoolers. J Fam Psychol. (2008) 22:30–40. doi: 10.1037/0893-3200.22.1.30. PMID: 18266530

[55] Gershoff ET Grogan-Kaylor A . Spanking and child outcomes: Old controversies and new meta-analyses. J Fam Psychol. (2016) 30:453–69. doi: 10.1037/fam0000191. PMID: 27055181 PMC7992110

[56] Hayes SC Luoma JB Bond FW Masuda A Lillis J . Acceptance and Commitment Therapy: Model, processes and outcomes. Behav Res Ther. (2006) 44:1–25. doi: 10.1016/j.brat.2005.06.006. PMID: 16300724

[57] Sheppes G Scheibe S Suri G Radu P Blechert J Gross JJ . Emotion regulation choice: A conceptual framework and supporting evidence. J Exp Psychol Gen. (2014) 143:163–81. doi: 10.1037/a0030831. PMID: 23163767

[58] Richards JM Gross JJ . Emotion regulation and memory: The cognitive costs of keeping one’s cool. J Pers Soc Psychol. (2000) 79:410–24. doi: 10.1037/0022-3514.79.3.410. PMID: 10981843

[59] Pennebaker J . Writing about emotional experiences as a therapeutic process. Psychol Sci. (1997) 8:162–6. doi: 10.1111/j.1467-9280.1997.tb00403.x. PMID: 41933073

[60] Morelen D Shaffer A Suveg C . Maternal emotion regulation: Links to emotion parenting and child emotion regulation. J Fam Issues. (2016) 37:1891–916. doi: 10.1177/0192513X14546720. PMID: 41930703

[61] Havighurst SS Wilson KR Harley AE Kehoe C Efron D Prior MR . Tuning into Kids”: Reducing young children’s behavior problems using an emotion coaching parenting program. Child Psychiatry Hum Dev. (2013) 44:247–64. doi: 10.1007/s10578-012-0322-1. PMID: 22820873

[62] Chan CKY Fu K Liu SKY . Incorporating emotion coaching into behavioral parent training program: Evaluation of its effectiveness. Child Psychiatry Hum Dev. (2024) 55:236–46. doi: 10.1007/s10578-022-01402-y. PMID: 35838816

[63] Baumeister RF Bratslavsky E Muraven M Tice DM . Ego depletion: Is the active self a limited resource? J Pers Soc Psychol. (1998) 74:1252–65. doi: 10.1037/0022-3514.74.5.1252. PMID: 9599441

