# Supplementary material for: Harsh discipline mediates the association between parenting stress and internalizing problems in children and adolescents: survey-based and online intervention evidence
Source: Front Psychiatry. 2026 Apr 30;17:1756447. doi: 10.3389/fpsyt.2026.1756447 (PMC13171506; doi:10.3389/fpsyt.2026.1756447)
Supplement: Supplementary file 1 [file Table1.docx]

***Supplementary Material***

**TABLE 1** Example of the intervention training courses in Study2.

| Stage | Course name | Course content | Course target |
| --- | --- | --- | --- |
| Day5 | Be a Good-Tempered Parent | Introduction to emotion regulation strategies | Intrapersonal ER |
|  |  | Emotional ABC Theory |  |
|  |  | Rain's four-step method for managing emotions |  |
|  |  | How to properly vent your negative emotions |  |
| Day9 | Parent-child interaction | Ways to improve parent-child relationships | Interpersonal ER |
|  |  | Common parent-child interactive games |  |
|  |  | Good parent-child communication skills |  |
| Day13 | Child development | Dealing with and responding to children's mistakes and misbehavior | Cognitive Reappraisal |
|  |  | Physical and psychological characteristics of children and adolescents |  |
|  |  | Cultivating good psychological qualities and lifestyle habits in children |  |
| Day17 | Family Parenting Style | Four types of parenting | Family systemic ER |
|  |  | Parenting causes of children's behavior |  |
|  |  | Creating a family environment that benefits parents and children |  |

Emotion regulation constitutes the theoretical core and applied logic underlying the entire intervention system. The curriculum design follows a progressive developmental pathway, advancing from parental intrapersonal emotion regulation to parental interpersonal emotion regulation.

The early phase of intervention (Day 5) focuses on intrapersonal emotion regulation, aiming to help parents master strategies to manage their own negative emotions. This stage serves as the foundation of the entire intervention, as parental emotional reactivity is often the direct trigger of inappropriate parenting behaviors. Building on this foundation, subsequent sessions are not isolated parenting-technique instruction but are systematically structured as interpersonal emotion regulation. Day 9 transfers emotion regulation skills to parent-child interaction contexts, emphasizing a shift from emotional neglect to emotional acceptance. This module reduces conflict and fosters connection through improved communication and shared activities. Day 13 targets cognitive reappraisal by helping parents understand developmental patterns and reframe child misbehavior, such as tantrums or defiance, as developmentally normal rather than intentional. This shift transforms disciplinary control into empathic guidance. Day 17 elevates regulation to the family system level, helping parents understand how their parenting style shapes child behavior and the family emotional climate. This module fosters a sustainable ecosystem of emotional regulation through family rules and emotional support.

The core objective is not to exert control over children's behavior but to guide parents in regulating children's emotional states through family rules, consistent enforcement, and emotional support. In this framework, enhanced parental intrapersonal regulation enables parents to become effective interpersonal emotional regulators for their children, thereby alleviating children's emotional distress. Viewed holistically, the four-phase curriculum structure forms a coherent continuum centered on emotional regulation as the core mechanism. It progresses from the individual to the interpersonal, from regulating oneself to regulating one's child, and from immediate strategies to systemic environments, demonstrating the continuous deepening of emotional regulation capabilities across different levels of intervention.
